# Supplementary figures and images for: AMPylation matches BiP activity to client protein load in the endoplasmic reticulum
Source: eLife. 2015 Dec 17;4:e12621. doi: 10.7554/eLife.12621 (PMC4739761; doi:10.7554/eLife.12621)

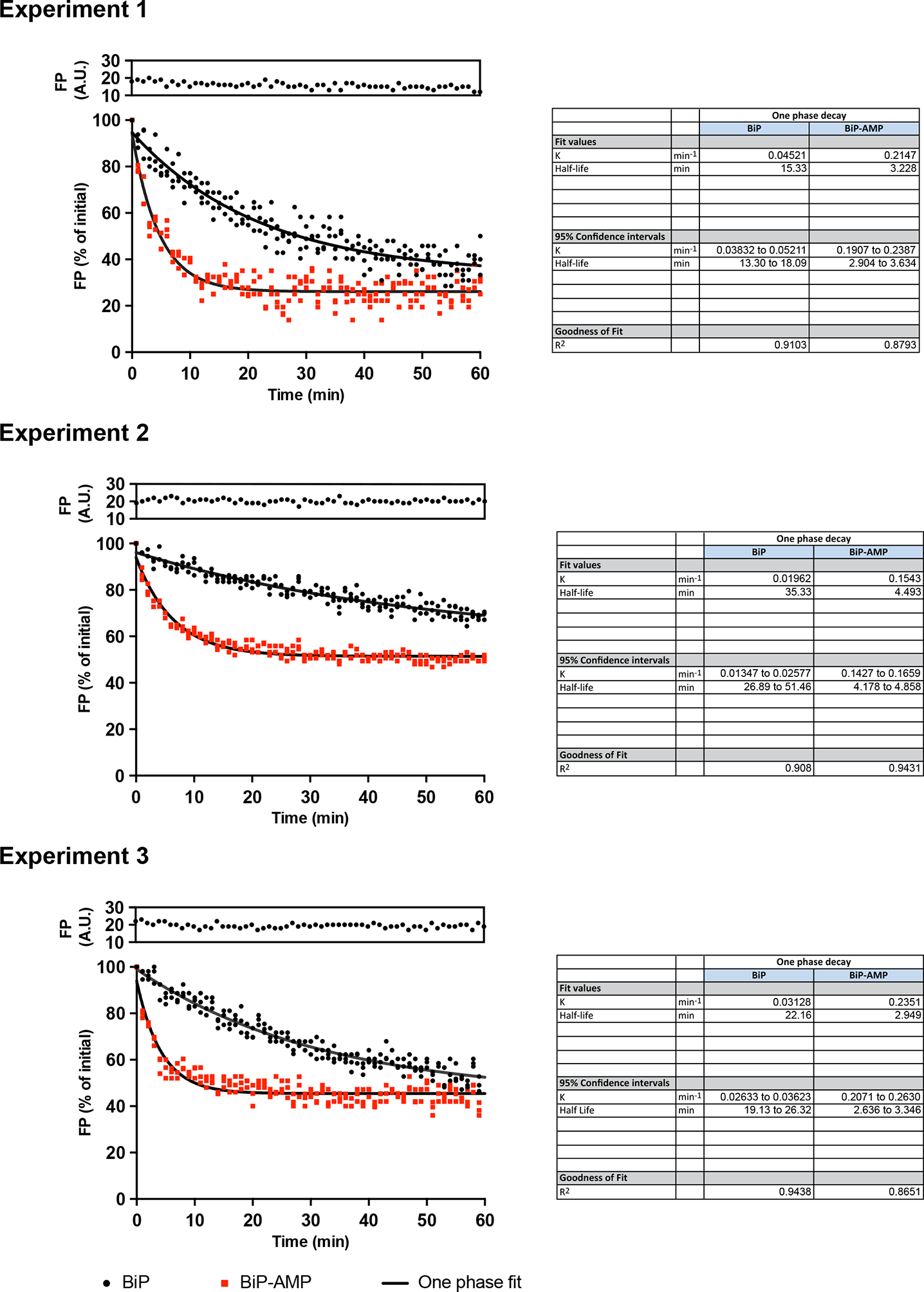

Supplement: Figure 8—source data 1. — The insert on top of each graph shows the absolute fluorescence polarization (FP) signals in arbitrary units (A.U.) of a reference sample containing only free fluorescent substrate peptide, which were used to create normalized FP traces of samples containing BiP + peptide. The initial values (after reference signal subtraction) were set to 100%. The fit to a single phase decay curve (tabulated here) was better than to a two phase model. The fit values from the three experiments were used to calculate the average values for “koff” and the half-lives. Experiment 3 is shown in Figure 8E. DOI: http://dx.doi.org/10.7554/eLife.12621.020 [file elife-12621-fig8-data1.zip › F8_E_source.tif]

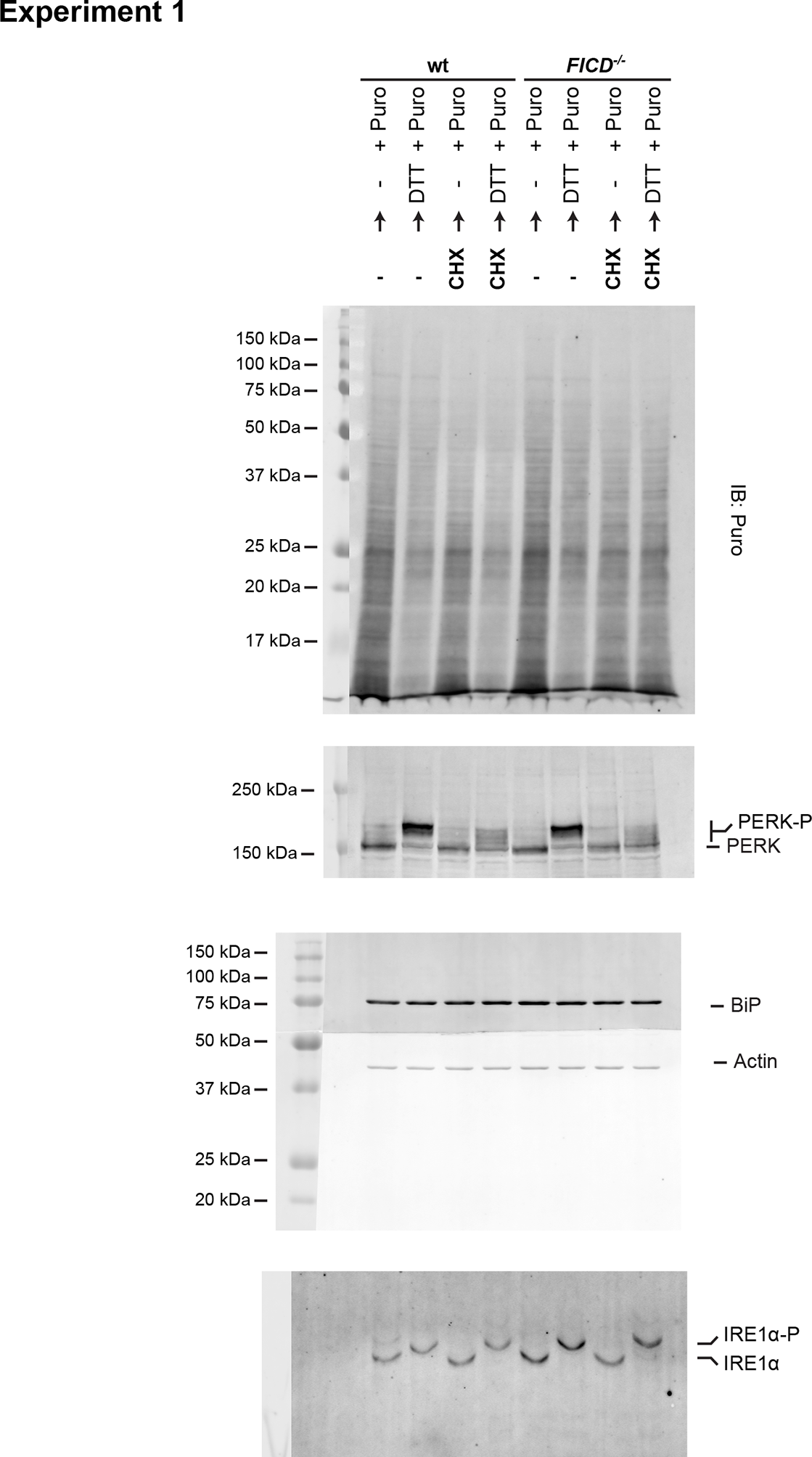

Supplement: Figure 10—source data 1. — DOI: http://dx.doi.org/10.7554/eLife.12621.024 [file elife-12621-fig10-data1.zip › F10_E_source.tif]

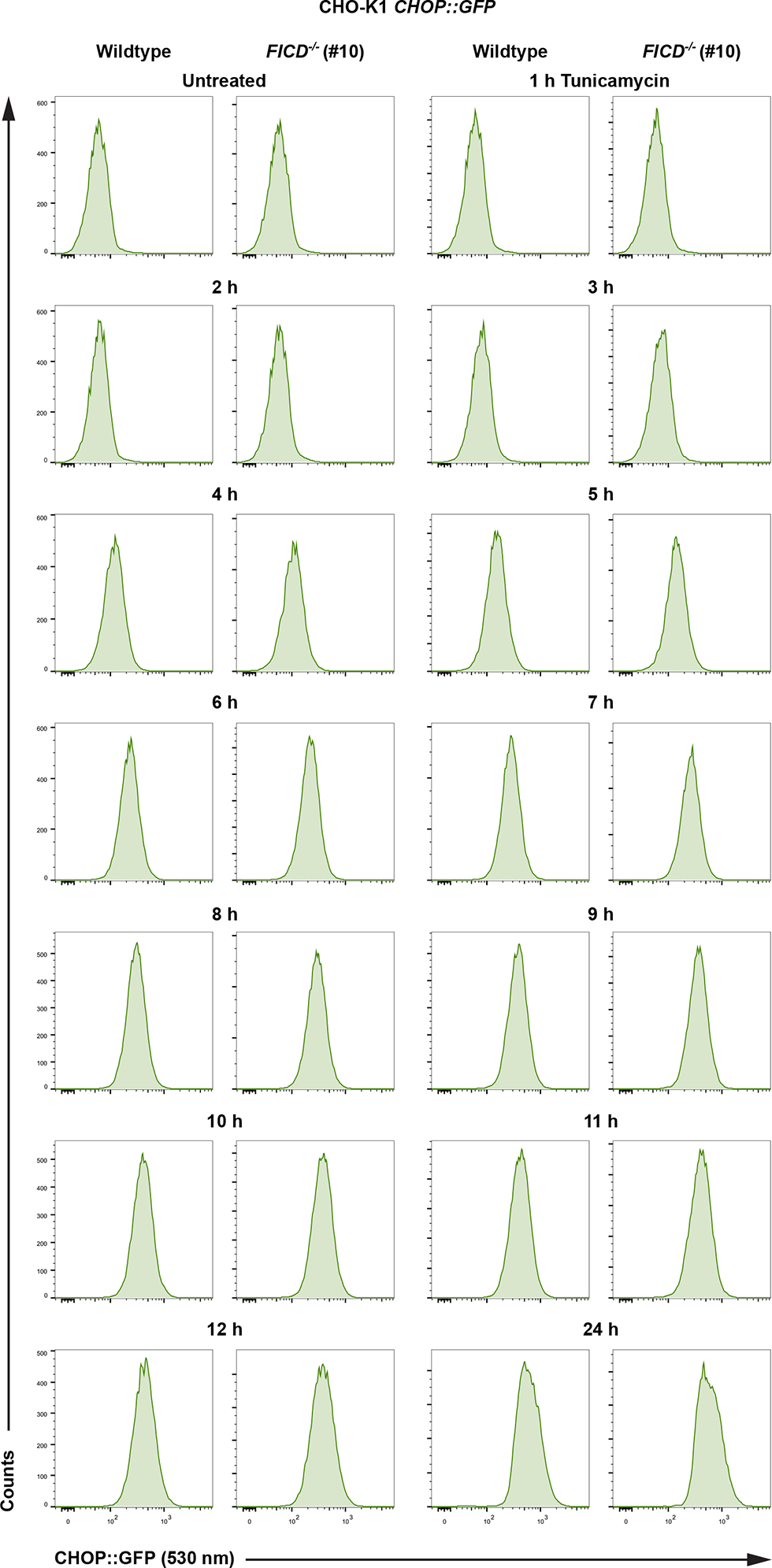

Supplement: Figure 10—source data 2. — DOI: http://dx.doi.org/10.7554/eLife.12621.025 [file elife-12621-fig10-data2.zip › F10S2_D_source.tif]
